# Pathogenesis of Human Papillomaviruses Requires the ATR/p62 Autophagy-Related Pathway

**DOI:** 10.1128/mBio.01628-20

**Published:** 2020-08-12

**Authors:** Shiyuan Hong, Yan Li, Paul J. Kaminski, Jorge Andrade, Laimonis A. Laimins

**Affiliations:** a Department of Microbiology-Immunology, Northwestern University, Feinberg School of Medicine, Chicago, Illinois, USA; b Center for Research Informatics, The University of Chicago, Chicago, Illinois, USA; Icahn School of Medicine at Mount Sinai

**Keywords:** DNA replication, GATA4, HPV, inflammation, interferons, p62

## Abstract

High-risk human papillomaviruses (HPVs) constitutively activate the ataxia telangiectasia and Rad3-related (ATR) DNA damage response pathway, and this is required for viral replication. In fibroblasts, activated ATR regulates transcription of inflammatory genes through its negative effects on the autophagosome cargo protein p62. In addition, suppression of p62 results in increased levels of the transcription factor GATA4, leading to cellular senescence. In contrast, in HPV-positive keratinocytes, we observed that activation of ATR resulted in increased levels of phosphorylated p62, which in turn lead to reduced levels of GATA4. Knockdown of ATR in HPV-positive cells resulted in decreased p62 phosphorylation and increased GATA4 levels. Transcriptome sequencing (RNA-seq) analysis of HPV-positive cells identified inflammatory genes and interferon factors as negative transcriptional targets of ATR. Furthermore, knockdown of p62 or overexpression of GATA4 in HPV-positive cells leads to inhibition of viral replication. These findings identify a novel role of the ATR/p62 signaling pathway in HPV-positive cells.

## Abstract (precis)

High-risk human papillomaviruses (HPVs) infect epithelial cells and induce viral genome amplification upon differentiation. HPV proteins activate the ATR DNA damage repair pathway, and this is required for HPV genome amplification. In the present study, we show that HPV-induced ATR activation also leads to suppression of expression of inflammatory response genes. This suppression results from HPV-induced phosphorylation of the autophagosome cargo protein p62 which regulates the levels of the transcription factor GATA4. Activation of p62 in normal fibroblasts results in senescence, but this is not seen in HPV-positive keratinocytes. Importantly, knockdown of p62 or overexpression of GATA4 in HPV-positive cells abrogates viral replication. This study demonstrates that activation of ATR in HPV-positive cells triggers a p62-directed pathway inducing suppression of inflammatory gene expression independent of DNA repair and facilitating HPV replication.

## INTRODUCTION

The DNA damage repair (DDR) pathway consists of a complex network of cell signaling and enzymatic factors which are activated in response to DNA damage. The two pathways that mediate the homologous recombination arm of DDR are the ataxia-telangiectasia mutated (ATM) kinase and the ataxia-telangiectasia and RAD3-related (ATR) kinase pathways. The ATM and ATR kinases are activated in response to double- or single-strand breaks as well as abnormal DNA structures that arise as a result of stalled or collapsed replication forks. This then leads to the phosphorylation and activation of a series of downstream effectors. A number of viruses, including human papillomaviruses (HPVs), simian virus 40 (SV40), and Epstein-Barr virus (EBV) utilize the DNA damage response for viral replication (1–3).

HPVs are small double-stranded DNA viruses that target stratified epithelia for infection. More than four hundred types of HPVs have been identified, and a subset of about 10 types, referred to as high-risk HPVs, are the causative agents of most cervical cancers and many oropharyngeal cancers (4–7). The most prominent of these are high-risk HPV16, HPV18, and HPV31 types. All HPVs infect cells in the basal epithelial layer and establish their genomes as extrachromosomal elements or episomes that replicate in synchrony with cellular replication. In contrast, productive HPV replication, which is referred to as amplification, occurs after differentiation in suprabasal layers (8). High-risk HPVs encode E6 and E7 oncoproteins as well as E1 and E2 proteins that control replication and gene expression (9). The E1̂E4 and E5 proteins as well as two capsid proteins L1 and L2 are expressed primarily in highly differentiated cells (10). Both the ATM and ATR DDR pathways are constitutively activated by high-risk HPV E1, E6, and E7 proteins alone independent of viral replication (11, 12).

Activation of the ATM pathway is necessary for HPV genome amplification, which is mediated by downstream effectors such as SMC1, NBS1, BRCA1, and RAD51 that are preferentially recruited to breaks in viral genomes at the expense of cellular sequences. Knockdown or inhibition of the enzymatic activities of any of these factors blocks HPV amplification (13–15). Similarly, high-risk HPV proteins induce ATR phosphorylation (16–18). This phosphorylation is dependent on binding of topoisomerase IIβ-binding protein 1 (TopBP1), the ATR-interacting protein (ATRIP) (19), and claspin (20). Once activated, ATR triggers the activation of downstream factors such as CHK1 (21), BRCA1 (22), and minichromosome maintenance (MCM) proteins (23), which leads to cell cycle exit or repair of DNA breaks. In contrast to the effect of ATM inhibition, suppression of the ATR pathway significantly impairs both HPV amplification and stable maintenance replication (16, 18, 24).

Although ATR has many reported functions, knowledge of how it regulates viral replication is limited. Most studies regarding ATR function focus on its role in initiation of DDR (25). ATR has also been implicated in regulating transcription through its effects on the transcriptional factor GATA4 (26), which can be degraded by the autophagy-related factor p62 (also known as sequestosome 1 [SQSTM1]). ATR has been reported to negatively regulate p62 in fibroblasts (26). Furthermore, treatment of cells with ATR inhibitors leads to decreased levels of the GATA4, resulting in changes in the expression of genes involved in senescence and inflammation (26). While it is known that ATR itself is important for HPV replication, it is unclear whether ATR functions through p62/GATA4 to regulate HPV replication.

In this study, we examined whether the transcriptional regulatory properties directed by ATR are critical for HPV replication, and if so which downstream targets are important. Using transcriptome sequencing (RNA-seq) and short hairpin RNA (shRNA) methods in HPV-positive cells, we observed that ATR knockdown increased expression of many inflammatory genes, such as interleukin 6 (IL-6), chemokine (C-X-C motif) ligand 2 (CXCL2), and CXCL10, as well as interferon kappa (IFN-κ). We observed that the levels of phosphorylated p62, but not total p62, were increased in HPV-positive cells, and that ATR was responsible for this phosphorylation. In contrast to previous studies in fibroblasts (26), our experiments indicated that ATR positively regulates p62 in HPV-positive cells and ATR knockdown reduces p62 activation with minimum effect on expression of other autophagy markers. As a consequence, GATA4 levels are low in HPV-positive cells and were increased by knockdown of ATR protein. Consistent with the effect of ATR knockdown, overexpression of GATA4 resulted in increased levels of inflammatory genes and IFN-κ in HPV-positive cells. Taken together, our studies reveal a novel feedback mechanism between ATR activation, p62 signaling, and the regulation of expression of GATA4-dependent genes during HPV life cycle.

## RESULTS

### Activation of the ATR pathway is necessary for HPV replication.

**FIG 1:**
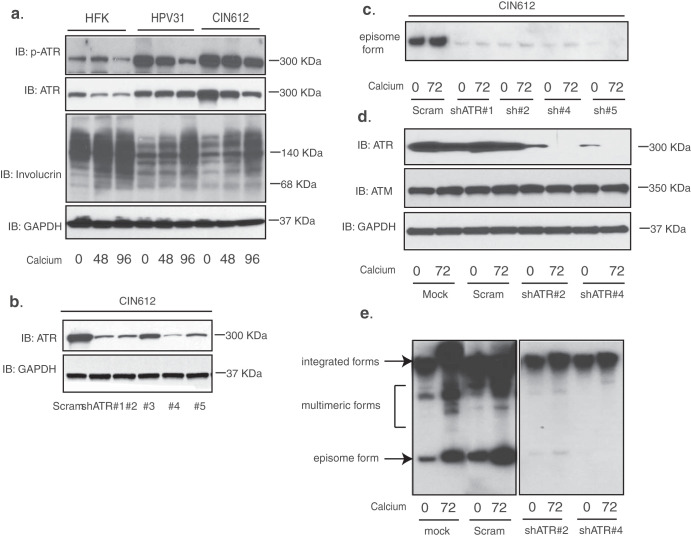
Knockdown of ATR in cells that maintain viral episomes blocks HPV episome maintenance and amplification upon keratinocyte differentiation. (a) Western blot analysis of p-ATR, ATR, involucrin, and GAPDH levels in HFKs, HPV31, and HPV31-positive CIN612 cells differentiated in high-calcium media for the indicated times (in hours). (b) Western blot analysis of ATR and GAPDH levels in CIN612 cells transiently transduced with five different shRNA lentiviruses against ATR. (c) Southern blot analysis for HPV31 episomes in CIN612 cells with transient ATR knockdown following differentiation in high-calcium media for the indicated times (in hours). (d). Western blot analysis of ATR, ATM, and GAPDH protein levels in mock, scrambled control, and two stably selected ATR knockdown CIN612 cells upon differentiation in high-calcium media for 72 h. (e) Southern blot analysis for HPV31 episomes in CIN612 cells with stable ATR knockdown following differentiation in high-calcium media for the indicated times (in hours). The two panels are from the same blot. All results are representative of observations from two or three independent experiments. IB, immunoblotting.

To investigate whether ATR’s ability to regulate gene expression is important for HPV pathogenesis, we first sought to confirm previous observations that constitutive ATR activation is critical for viral replication. In our initial studies, treatment of HPV-positive cells with inhibitors of ATR or CHK1 resulted in loss of viral episomes and the treated cells contained almost exclusively integrated genomes (18). To investigate the effects of ATR’s transcriptional ability in HPV-positive cells, we first examined by Western blot analysis the levels of total and phosphorylated ATR in normal human foreskin keratinocytes (HFKs), human keratinocytes that stably express HPV31 episomes (HPV31), and CIN612 cells that were derived from a HPV31-positive biopsy specimen. Our data showed that the total levels of ATR as well as phosphorylated ATR (p-ATR) were increased in HPV31 cells and CIN612 cells. Similar to control cells, p-ATR levels decreased modestly upon differentiation but remained higher than in HFKs (Fig. 1a). This is consistent with recent studies showing that ATR is constitutively activated in HPV-positive cells (16, 18). We next transiently knocked down ATR using shRNA lentiviruses in HPV-positive CIN612 cells (Fig. 1b), followed by differentiation in high-calcium media for an additional 72 h. Southern blot analysis of these cells showed that ATR knockdown suppressed maintenance of viral episomes (Fig. 1c). The loss of episomes also prevented viral amplification. The transiently transduced CIN612 cells were then selected for puromycin resistance, and stable knockdown lines were isolated using the two most efficient sets of shRNAs. The stable cells were assayed by Western blot analysis and showed severely decreased levels of ATR but not of ATM (Fig. 1d). Southern blot analysis of these lines revealed abrogation of both episomal maintenance in undifferentiated cells and amplification upon differentiation (Fig. 1e), which is consistent with previous findings (18). Cells with stable ATR knockdowns contained primarily integrated copies of HPV genomes with minimal episomes (Fig. 1e, right panel). Furthermore, these stable ATR knockdown HPV-positive cells continued to proliferate for more than 15 passages with no noticeable growth defects.

### Suppression of ATR activation by shRNA knockdown increases expression of sets of inflammatory genes in HPV-positive cells.

**FIG 2:**
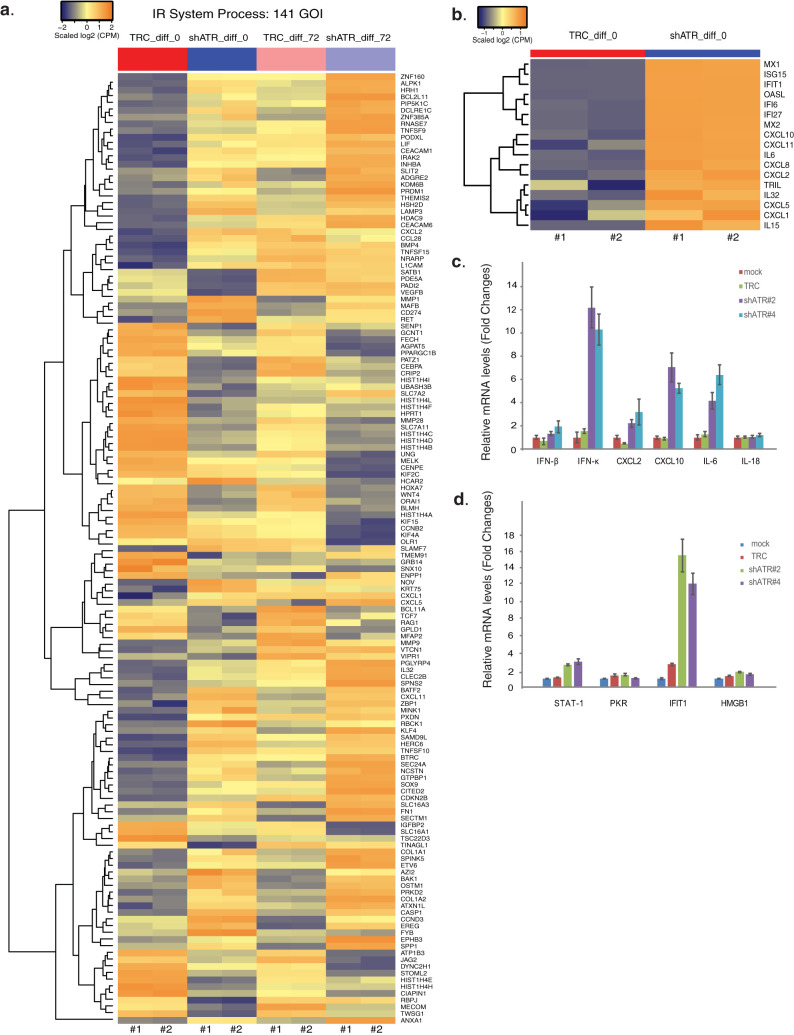
Downstream targets of ATR transcriptional regulation in HPV-positive CIN612 cells. (a) The heat map of genes of the immune response system process pathway in the scrambled (TRC) and ATR knockdown (shATR) cells with or without differentiation (72 h). (b) Heat maps of highly differentially expressed genes modulated by ATR knockdown. (c) RT-PCR analysis of IFN-β, IFN-κ, CXCL2, CXCL10, IL-6, and IL-18 in CIN612 cells with ATR knockdown. (d) RT-PCR analysis of STAT-1, PKR, IFIT1, and HMGB1 in CIN612 cells with ATR knockdown. GAPDH was used as an internal control and for normalization of the data. Data are means ± standard errors (error bars). *P* value <0.05. All results are representative of observations from two or more independent experiments.

We next investigated whether ATR affected transcription of any genes in HPV-positive cells by performing an unbiased global RNA-seq analysis using stable ATR knockdown CIN612 cells. Both ATR knockdown and scrambled control cells were differentiated in high-calcium media for 72 h, and RNAs were extracted for RNA-seq analysis. To identify genes regulated by ATR, a twofold change with a false discovery rate (FDR)-corrected *P* value of 0.05 was set as the cutoff with a minimum of 40 to 45 reads for the lowest signature detection level. Any differentially expressed genes which were regulated by ATR were used to construct a functional enrichment analysis via Ingenuity Pathway Analysis (IPA) tools. From this analysis, we found genes enriched in various pathways such as immune response and cell cycle (see Table S1 in the supplemental material). To show the effect of ATR on gene expression of immune response, the changes in expression of a subset of genes belonging to the immune response system process pathway were shown in Fig. 2a for both undifferentiated and differentiated cells. The changes in gene expression between the knockdown (shATR) cells and the scrambled control (TRC) cells were substantial and increased upon epithelial differentiation. A list of a subset of immune response genes (also known as antiviral or inflammatory genes) exhibiting prominent changes was shown in Fig. 2b. This includes several interferon-stimulated genes (ISGs), interleukins, and CXCL chemokines. The full set of genes altered in these ATR knockdown cells can be found at the website described in Materials and Methods. We then confirmed the RNA-seq data for a subset of inflammatory genes by reverse transcription-PCR (RT-PCR) analysis using undifferentiated ATR knockdown CIN612 cells (Fig. 2c). In this analysis, we included IL-18 and IFN-κ, as IL-18 is considered a major inflammatory gene, while IFN-κ is specifically expressed in skin and was recently shown to suppress HPV replication (27). Of note, the RT-PCR studies showed that ATR knockdown greatly increased the expression of IFN-κ, but not IFN-β or high mobility group box 1 (HMGB1), which is known as a proinflammatory cytokine induced by type II interferons (28) (Fig. 2c and d). Furthermore, expression of STAT-1 or protein kinase R (PKR), which are known to be regulated by IFN-β, were not altered by ATR knockdown (Fig. 2d). In contrast, the levels of IFIT1, another ISG that interacts with HPV E1 protein, as well as expression of IL-6, CXCL2, and CXCL10 were altered in the ATR knockdown cells (Fig. 2c). This indicates that ATR targets expression of a subset of inflammatory response genes and IFN-κ.

10.1128/mBio.01628-20.3 TABLE S1 Target pathways of ATR transcriptional regulation by IPA analysis in HPV-positive CIN612 cells. The scrambled (TRC) and two individual ATR knockdown (shATR#2 and shATR#4) cells with or without differentiation (diff_72 and diff_0 hrs) are processed for RNA-seq, followed by IPA analysis for ATR downstream pathways. The four comparisons are as follows: comparison 1 (comp 1), shATR#2_diff_0 versus TRC_diff_0; comp 2, shATR#4_diff_0 versus TRC_diff_0; comp 3, shATR#2_diff_72 versus TRC_diff_72; comp 4, shATR#4_diff_72 versus TRC_diff_72. Download Table S1, XLSX file, 0.1 MB. Copyright © 2020 Hong et al. 2020 Hong et al. This content is distributed under the terms of the Creative Commons Attribution 4.0 International license.

### ATR phosphorylates p62 in HPV-positive cells.

**FIG 3:**
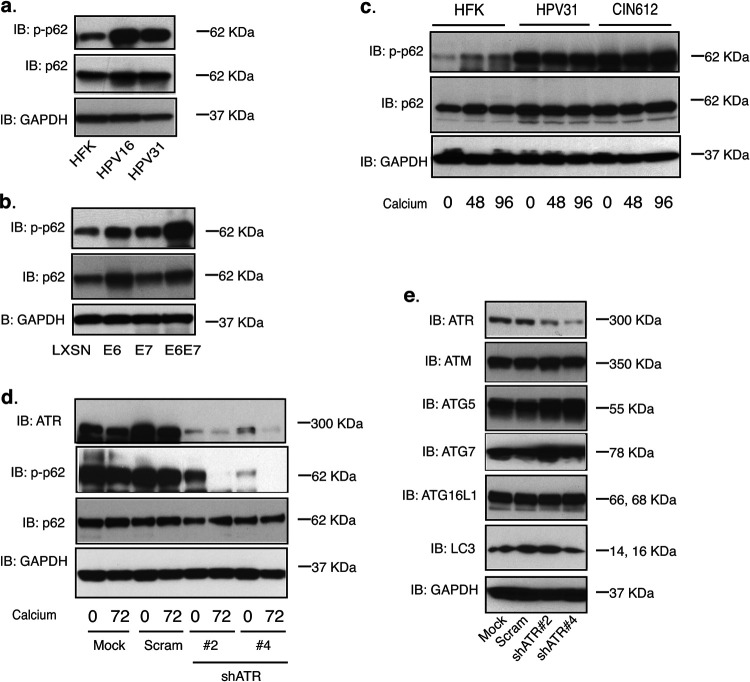
ATR phosphorylates p62 in HPV-positive CIN612 cells. (a) Western blot analysis of p-p62, p62, and GAPDH levels in HFK, HPV16, and HPV31 cells. (b) Western blot analysis of p-p62, p62, and GAPDH levels in HFK cells expressing LXSN vector, HPV31E6, HPV31E7, and HPV31E6/E7. (c) Western blot analysis of p-p62, p62, and GAPDH levels in HFK, HPV31, and HPV31-positive CIN612 cells differentiated in high-calcium media for the indicated times (in hours). (d) Western blot analysis of ATR, p-p62, p62, and GAPDH protein levels in mock, shRNA control, and two different sets of ATR shRNA lentivirus-infected CIN612 cells upon differentiation in high-calcium media for 72 h. (e) Western blot analysis of ATR, ATM, ATG5, ATG7, ATG16L1, LC3, and GAPDH protein levels in mock, shRNA control, and two different sets of ATR shRNA lentivirus-infected CIN612 cells. All results are representative of observations from two or three independent experiments.

It was next important to investigate the mechanism underlying why ATR knockdown increased expression of genes involved in inflammation and IFN regulation in HPV-positive cells. Previous studies reported that selective autophagy suppresses expression of senescence-associated secretory phenotype (SASP) genes such as IL-6, IL-8, and CXCL1 in a GATA4-dependent manner through the action of p62 (26). We therefore examined whether ATR could regulate the autophagy factor p62. In HPV-positive keratinocytes that maintain HPV16 or HPV31 episomes, the phosphorylated p62 levels were substantially increased in comparison with those in HFKs (Fig. 3a), while the total p62 levels were found to be modestly increased in HPV-positive keratinocytes compared to HFKs. To determine which viral protein was responsible for this increase, cell lines generated by infection of HFKs with retroviruses expressing either E6, E7, or both were used to evaluate phosphorylated p62 levels by Western blot analysis. Figure 3b indicates that both HPV31 E6 and E7 proteins were responsible for induction of p62 phosphorylation and act synergistically. In addition, E6 slightly increased the levels of total p62. The total p62 protein levels were similar in HFKs, HPV31, and CIN612 cells, while only the phosphorylated levels were increased (Fig. 3c). To confirm that ATR acts upstream of p62 phosphorylation, we examined the levels of phospho-p62 by Western blot analysis using the stable ATR knockdown HPV-positive cells generated as described above in the legend to Fig. 1d. Our studies showed that knockdown of ATR in HPV-positive cells significantly reduced ATR levels and consequently diminished p62 phosphorylation (Fig. 3d). In addition, ATR knockdown did not affect other autophagy markers, as the levels of ATG5, ATG7, ATG16L1, and LC3 were unchanged from control cells (Fig. 3e).

### Knockdown of p62 blocks HPV genome maintenance as episomes.

**FIG 4:**
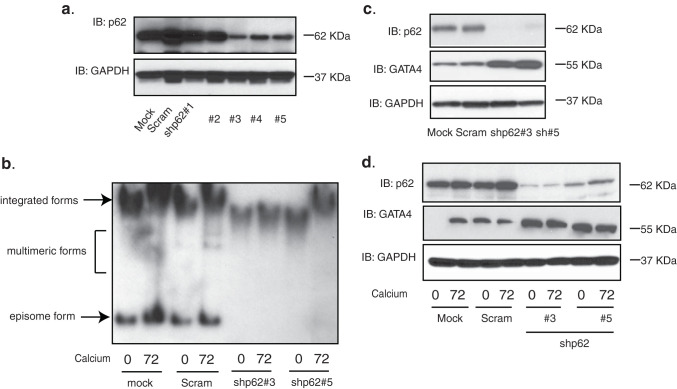
Suppression of p62 inhibits HPV genome amplification and increases GATA4 expression in CIN612 cells. (a) Western blot analysis of p62 and GAPDH levels in CIN612 cells transiently transduced with five different shRNA lentiviruses against p62. (b) Southern blot analysis for HPV31 episomes in CIN612 cells with stable p62 knockdown following differentiation in high-calcium media for the indicated times (in hours). (c) Western blot analysis of p62, GATA4, and GAPDH protein levels in mock, scrambled control, and two stably selected p62 knockdown CIN612 cells. (d) Western blot analysis of p62, GATA4, and GAPDH protein levels in the cells described in the legend for panel c following differentiation in high-calcium media for the indicated times (in hours). In the lane of mock cells at 0 h of differentiation, there was likely a bubble over the GATA4 band that occurred during the transfer process. The presence of GATA4 in undifferentiated cells is also shown in Fig. 5c. All results are representative of observations from two or three independent experiments.

We next investigated whether p62 was important for maintenance of HPV episomes as well as amplification. CIN612 cells were transduced with a series of lentiviral shRNAs against p62, and Western blot analysis demonstrated that three out of five were effective in reducing p62 levels (Fig. 4a). The selected stable knockdown lines were then differentiated in high-calcium media and assayed for HPV episomes by Southern blot analysis. Figure 4b showed that the levels of HPV episomes increased upon differentiation in the mock infected cells and the cells expressing a scrambled shRNA control. In contrast, in cells in which p62 levels were suppressed by shRNAs, the maintenance of HPV episomes was lost, and cells contained almost exclusively integrated copies. Lysates of p62 knockdown cells were also assayed by Western blot analysis for the levels of GATA4 and glyceraldehyde-3-phosphate dehydrogenase (GAPDH). The p62 levels in cells expressing shRNAs were reduced in comparison to the mock or scrambled cells (Fig. 4c), and correspondingly, an increase in GATA4 protein levels was observed. Upon epithelial differentiation, p62 levels were found to be high in the mock or scrambled cells, while only low levels were observed in the p62 knockdown cells (Fig. 4d). Consistently, increased levels of GATA4 were seen in undifferentiated or differentiated p62 knockdown cells. This confirms that p62 negatively regulates GATA4 stability in HPV-positive cells.

### GATA4 overexpression upregulates expression of inflammatory genes in HPV-positive cells.

**FIG 5:**
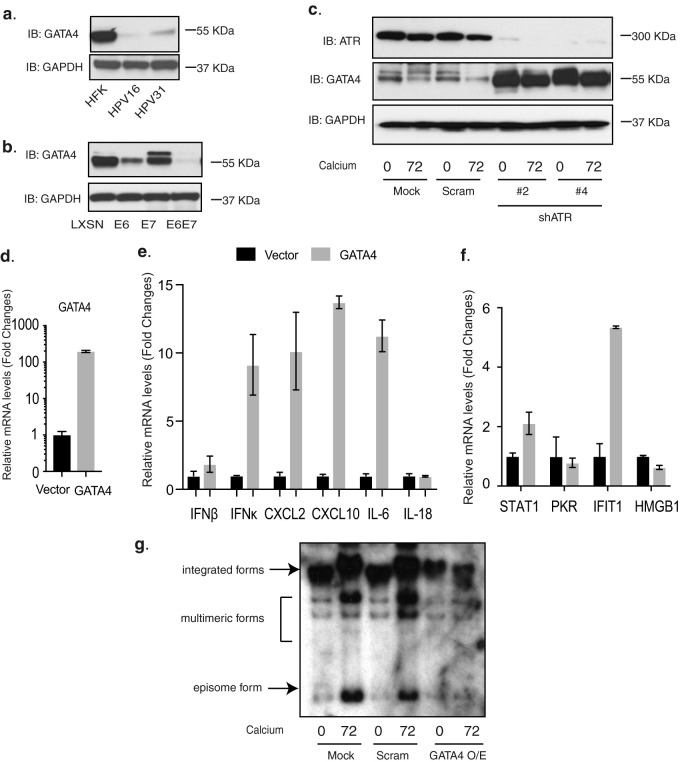
Overexpression of GATA4 increases expression of inflammatory genes and IFN-κ in CIN612 cells. (a) Western blot analysis of GATA4 and GAPDH levels in HFK, HPV16, and HPV31 cells differentiated in high-calcium media for the indicated times (in hours). (b) Western blot analysis of GATA4 and GAPDH levels in HFK cells expressing LXSN vector, HPV31E6, HPV31E7, and HPV31E6/E7. (c) Western blot analysis of ATR, GATA4, and GAPDH levels in mock, scrambled control, and two stably selected ATR knockdown CIN612 cells upon differentiation in high-calcium media for 72 h. (d) RT-PCR analysis of GATA4 expression levels in CIN612 cells. (e) RT-PCR analysis of IFN-β, IFN-κ, CXCL2, CXCL10, IL-6, and IL-18 in GATA4-overexpressing CIN612 cells. (f) RT-PCR analysis of STAT-1, PKR, IFIT1, and HMGB1 in GATA4-overexpressing CIN612 cells. GAPDH was used as an internal control and for normalization of the data. Data are means ± standard errors. *P* value <0.05. (g) Southern blot analysis of HPV genome status in GATA4-overexpressing cells. Overexpression of GATA4 in HPV-positive cells leads to loss of episomes and cells with almost exclusively integrated genome copies (far two right lanes). All results are representative of observations from two or more independent experiments.

Our studies indicated that p62 knockdown increased GATA4 expression in HPV-positive cells (Fig. 4). Interestingly, the levels of phosphorylated p62 (p-p62) were substantially increased in HPV-positive cells, while total levels were only slightly increased (Fig. 3). Furthermore, the levels of GATA4 in HFKs stably maintaining HPV16 or HPV31 episomes were very low in comparison with HFKs (Fig. 5a). To test whether HPV E6 or E7 was responsible for this suppression, HFKs transduced with retroviruses expressing HPV31 E6, E7, or E6/E7 were assayed using Western blot analysis for GATA4 expression. The expression of E6 in HFKs reduced the expression levels of GATA4, while the expression of E7 had a moderate effect (Fig. 5b). The combination of E6 and E7 was most effective in reducing levels of GATA4, which likely occurs at the posttranslational level given the known activities of p-p62 (29, 30).

We next investigated whether GATA4 was responsible for ATR-dependent regulation of inflammatory gene expression in HPV-positive cells. We first confirmed that GATA4 levels were regulated by ATR in HPV-positive cells by examining ATR knockdown cells as described above. The ATR knockdown cells were differentiated in high-calcium media for 72 h and then assayed for GATA4 expression by Western blot analysis. Figure 5c showed that the levels of GATA4 in ATR knockdown cells were increased, and this was maintained upon epithelial differentiation. This analysis was obtained using the same set of cells as described in the legend to Fig. 1d. To test whether GATA4 regulates expression of the inflammatory genes that we observed to be controlled by ATR, GATA4 was overexpressed using flag-tagged GATA4-containing lentivirus in CIN612 cells that express high levels of ATR but low levels of GATA4. The stably transduced cells were then selected, isolated, and assayed by RT-PCR to confirm GATA4 overexpression (Fig. 5d). These cells were passaged more than 5 times with no defects in growth. In the GATA4-overexpressing cells, the expression of CXCL2, CXCL10, IL-6, and IFN-κ but not IFN-β, IL-18, or HMGB1 was increased (Fig. 5e and f). Consistent with the lack of an effect with IFN-β, expression of STAT-1 and PKR, which can be induced by IFN-β, was not significantly changed by GATA4 overexpression. In contrast, IFIT1 levels were increased in HPV-positive cells as a result of GATA4 overexpression (Fig. 5f). In addition, HMGB1 was not affected by GATA4 overexpression. Taken together, GATA4 overexpression in HPV-positive cells increased expression of a set of inflammatory genes as well as IFN-κ. Finally, we investigated whether stable overexpression of GATA4 affected episomal maintenance as well as amplification (Fig. 5g). Southern analysis of control and GATA4-overexpressing cells demonstrated a loss of HPV episomes with cells containing almost exclusively integrated genomes and no amplification. We concluded that GATA4 overexpression in HPV-positive cells also leads to loss of the ability to maintain viral episomes and amplify genomes.

## DISCUSSION

ATR is a critical DNA damage effector that induces DNA damage repair and checkpoint signaling upon genomic stress. Most studies focus on the role of ATR in DNA damage repair, but less is known about ATR’s other functions. Recent evidence indicates that ATR inhibition leads to altered expression of a variety of genes, including those regulating the immune response. HPV proteins alone have been shown to constitutively activate the ATR pathway, and this is necessary for both viral episomal maintenance in persistently infected undifferentiated cells and HPV amplification upon differentiation (18). We now show that in HPV-positive cells ATR activation suppresses expression of genes in immune response pathways, including many inflammatory genes, as well as IFN-κ. This activity is important for HPV viral replication and may also contribute to progression to cancer. The mechanism behind this regulation of transcription by ATR is through the phosphorylation of the autophagy factor p62 which in turn controls the stability of its binding partner, the transcriptional factor GATA4. In HPV-positive cells, ATR activation triggers p62 phosphorylation, resulting in a reduction in GATA4 levels. The ectopic expression of GATA4 in HPV-positive cells leads to increased expression of IL-6, CXCL2, CXCL10, as well as IFN-κ, and similar effects are seen following ATR knockdown in HPV-positive cells. The negative regulation of GATA4 by ATR in HPV-positive cells is in contrast to effects seen in fibroblasts where inhibition of ATR leads to GATA4 reduction (26). The reason for this difference is not clear but is most likely due to the effects of the multiple other cellular pathways modified by E6 and E7 which are the primary mediators of HPV-induced negative regulation of GATA4. Our studies indicate that HPV proteins differentially regulate the ATR/p62/GATA4 pathway to promote HPV replication along with the expression of immune regulatory factors.

GATA4 is a transcription factor that plays an important role in SASP as well as the inflammatory response (26). SASP is a mechanism by which senescent cells exert effects through secretion of proinflammatory cytokines, chemokines, and growth factors. In contrast to observations of fibroblasts where ATR activation results in increased levels of GATA4 leading to SASP, in HPV-positive keratinocytes, the opposite effect is seen with decreased levels of GATA4. Expression of E6 in conjunction with E7 leads to a decrease in GATA4 protein levels, and this is mediated through the effects of ATR on p62. Our studies show that E6 and E7 are the primary effectors of the ATR-directed negative regulation of GATA4, but we cannot exclude the possibility that other viral factors such as E1 or E2 could further augment these effects. The expression of E6 and E7 allows for cells to overcome any effects due to ATR inhibition and remain proliferative. We previously reported that HPV-positive cells either treated with inhibitors of ATR or CHK1 had no effect on growth (18, 31).

Overexpression of GATA4 in HPV-positive cells greatly increases expression of IL-6, CXCL2, CXCL10, and IFN-κ, which suggests that ectopic GATA4 expression can induce the proinflammatory response. These observations are consistent with ATR knockdown experiments showing that ATR inhibition increases gene expression of IL-6, CXCL2, CXCL10, and IFN-κ. HPV-positive cells that either stably maintain viral episomes or express only the E6 and E7 viral oncoproteins do not undergo senescence as occurs in other cell types where ATR activation results in SASP through GATA4 induction. Furthermore, high-risk HPVs appear to be capable of preventing senescence even when ATR is knocked down, as these cells were still proliferative. Taken together, GATA4 modulates the inflammatory response in HPV-positive cells, and suppression of GATA4 by p62 is important for establishment of persistent HPV infection.

Activation of ATR can, in some cases, lead to the induction of autophagy, and p62 is a central member of this pathway (32). HPV proteins activate the ATR pathway, and the total p62 levels in HPV-positive cells are similar to those seen in HFKs, and only the phosphorylated forms are increased. While ATR phosphorylates p62 in HPV-positive cells, knockdown has no effect on expression of other autophagy protein markers such as LC3 and ATG7. This is in contrast to a recent report suggesting that HPV16 E6/E7 activate autophagy in SiHa or Caski cells (33), while another study showed that the presence of HPV16 E6/E7 negatively affects the autophagic process (34). In addition, overexpression of HPV16 E7 in normal human keratinocytes increases the levels of autophagy marker LC3-II (26). Our studies indicate that phosphorylated p62 has additional functions beyond autophagy and is consistent with studies from the Komatsu group showing that p62 phosphorylation induces NRF2 activation, contributing to the growth of human hepatocellular carcinomas (29). In addition, elevated phosphorylated p62 levels are associated with decreased immunoreactivity in amyotrophic lateral sclerosis and Alzheimer’s disease tissues (35).

ATR knockdown increases expression of many ISGs in HPV-positive cells, but this is not due to higher levels of IFN-β. Importantly, IFN-β expression was not changed in ATR knockdown HPV-positive cells. The expression of two ISGs STAT-1 and PKR, which can be stimulated by IFN-β, were also not altered. This indicates that IFN-β is not a transcriptional target of ATR. Interestingly, ATR knockdown or GATA4 overexpression leads to enhanced expression of IFIT1, but not PKR or HMGB1. IFIT1 has been shown to inhibit HPV E1 helicase activity to limit HPV viral replication (36), so GATA4 repression may be critical to suppressing this activity. The increase in IFIT1 expression in ATR knockdown cells or GATA4-expressing cells suggests that IFIT1 can be induced by cellular factors other than IFN-β, possibly IFN-κ. This idea is supported by the studies of the Stubenrauch group (37) which along with other groups showed that HPV suppresses IFN-κ expression (38–40). The reason suppression of IFN-κ expression is required for HPV replication is, however, not well understood. One explanation could be that HPV proteins repress IFN-κ transcription to decrease expression of pathogen pattern receptors along with other antiviral genes (37). Additional studies have suggested that IFN-κ decreases HPV viral transcription and replication in an sp100-dependent manner (27). Thus, it appears that HPV induces ATR activation, in part, to reduce IFN-κ levels that can suppress viral replication.

**FIG 6:**
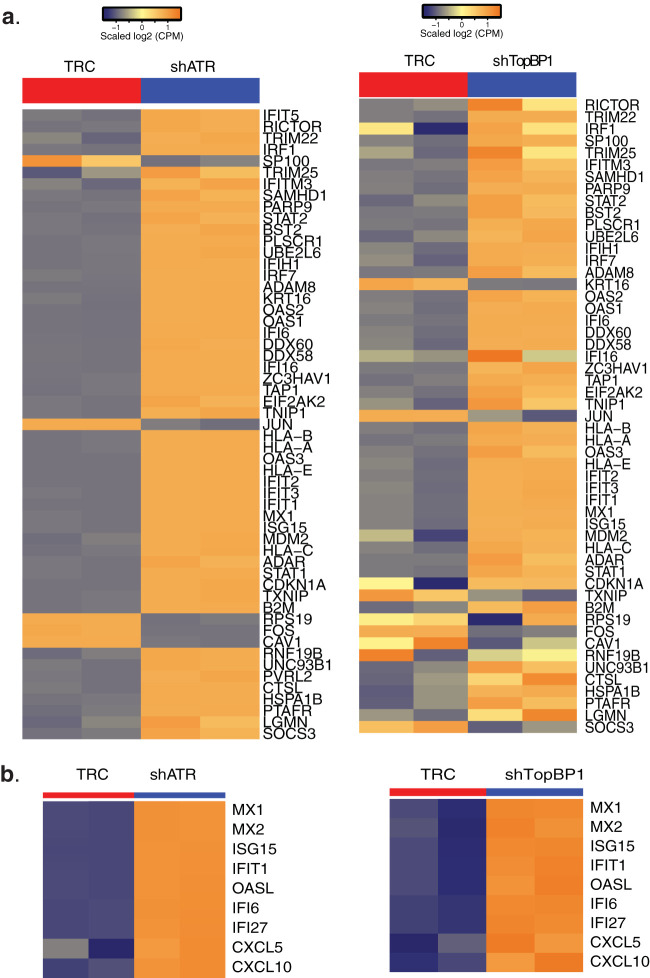
ATR and TopBP1 target common sets of differentially expressed genes in the immune response system response pathway in HPV-positive cells. (a) Heat map of a subset of genes of the immune response system response pathway in the scrambled (TRC) and ATR knockdown (shATR) cells versus TRC and TopBP1 knockdown (shTopBP1) cells, upon epithelial differentiation (72 h). (b) Heat maps of highly differentially expressed genes modulated by ATR knockdown or TopBP1 knockdown. All results are representative of observations from two or more independent experiments.

Our previous studies showed that TopBP1 levels are significantly increased in HPV-positive cells and that knockdown blocks HPV amplification (18). HPV-positive cells with either ATR or TopBP1 knockdown (24) remain proliferative and do not become growth arrested. We screened downstream targets of TopBP1 by RNA-seq approaches and found a number of potential downstream targets related to immune response (24), which are also detected in ATR knockdown cells (Fig. 6a). The patterns of several ISGs, including MX1, MX2, ISG15, IFIT1, OASL, IFI6, and IFI27, and two CXCL chemokines, namely, CXCL5 and CXCL10, are similar for both knockdowns (Fig. 6b). Furthermore, the effects of ATR knockdown on expression of inflammatory genes, such as IL-6, CXCL2, CXCL10, and IFN-κ, were also seen in TopBP1 knockdown cells (24). This suggests that the ATR pathway regulates expression of these immune-related genes. TopBP1 also acts as a transcription factor, which is dependent upon its phosphorylation, but this is independent of ATR (41). Our previous studies indicate that HPV proteins can induce TopBP1 phosphorylation in an AKT-dependent manner to regulate many signaling pathways such as blood vessel development and regulation of cell motility (24), which are not controlled by ATR. The Metascape online tools showed the top 20 pathways regulated by ATR in Fig. S1 in the supplemental material. TopBP1 thus targets different sets of downstream genes that are not regulated by ATR. Even for the same subset of genes in the immune effector process pathway, the effects of TopBP1 are quite distinct from those of ATR (Fig. S2). Thus, TopBP1 acts through at least two pathways to regulate gene expression. One involves activation of ATR, leading to p62 phosphorylation and GATA4 repression, and the second way is by AKT-mediated phosphorylation of TopBP1-activating genes such as E2F1 and p73.

10.1128/mBio.01628-20.1 FIG S1 Comparison of top 20 downstream pathways regulated by TopBP1 versus ATR. (a and b) The TopBP1-dependent genes (a) or ATR-dependent genes (b) identified by RNA-seq were categorized based on their functions and grouped using Metascape online tools. The top 20 pathways regulated by TopBP1 or ATR were selected and represented as bar figures. Download FIG S1, TIF file, 1.8 MB. Copyright © 2020 Hong et al. 2020 Hong et al. This content is distributed under the terms of the Creative Commons Attribution 4.0 International license.

10.1128/mBio.01628-20.2 FIG S2 TopBP1 also impacts downstream targets differentially than ATR in HPV-positive cells. The heat map shows patterns of differentially expressed genes belonging to the immune response system response pathway in the scrambled (TRC) and ATR knockdown (shATR) cells versus TRC and TopBP1 knockdown (shTopBP1) cells following a 72-hour differentiation in high-calcium media. All results are representative of observations from two or more independent experiments. Download FIG S2, TIF file, 2.9 MB. Copyright © 2020 Hong et al. 2020 Hong et al. This content is distributed under the terms of the Creative Commons Attribution 4.0 International license.

**FIG 7:**
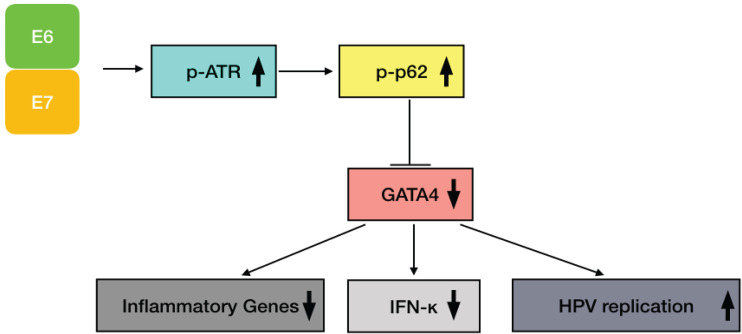
A model for activation of the ATR/p-p62/GATA4 pathway by HPV to regulate expression of inflammatory genes and HPV replication. HPV E6 and E7 activate ATR, which then phosphorylates p62 that in turn induces GATA4 degradation. This results in suppression of expression of inflammatory genes and IFN-κ as well as promotion of HPV replication.

In summary, we have identified a feedback loop mechanism between the ATR DNA damage response and immune signaling in the life cycle of HPVs (Fig. 7). In HPV-positive cells, ATR can repress expression of genes in the inflammatory response as well as IFN-κ through p62 phosphorylation without inducing autophagy. Importantly, knockdown of p62 also decreases HPV genome maintenance and amplification. The loss of either p62 or ATR results in increased levels of GATA4, a transcription factor that plays an important role in regulation of expression of inflammatory genes and IFN-κ. These findings suggest that ATR activation is important not only for HPV viral replication but that it also may be targeting the inflammatory response to allow for persistent HPV infection.

## MATERIALS AND METHODS

### Cell culture.

Normal human foreskin keratinocytes (HFKs) were isolated from neonatal foreskins as previously described (42). HFKs were cultured in E medium supplemented with epidermal growth factor (EGF) and cocultured with mitomycin-treated NIH 3T3 J2 fibroblasts. To make HPV16 and HPV31 cell lines that maintain viral episomes, HFKs were transfected with HPV genomes together with plasmids carrying genes that encoding drug resistance, and stable lines were selected. To make E6- or E7-expressing cells, HFKs were transduced with retroviruses expressing E6 or E7 as previously described (43). CIN612 cells that stably maintain HPV31 episomes are derived from a cervical intraepithelial neoplasia (CIN) II biopsy specimen. To induce differentiation, HPV-positive cells were cultured in keratinocyte basal medium without supplements and containing 1.5 mM CaCl_2_ for up to 96 h as described previously (43).

### Antibodies and Western blot analysis.

The antibodies used in this study were as follows: anti-involucrin and anti-GAPDH (Santa Cruz, Santa Cruz, CA); anti-ATR, anti-p-ATR, anti-ATM, anti-p-p62, anti-GATA4, and Autophagy Antibody Sampler kit, including ATG5, ATG7, ATG16L1, and LC3 (Cell Signaling, Danvers, MA); anti-p62 (American Research Products, Waltham, MA); and anti-p53 (Sigma, St. Louis, MO). Cell lysates were extracted and assayed by Western blot analysis as previously described (43). Briefly, keratinocytes were separated from J2 feeders by Versene (phosphate-buffered saline [PBS] containing 0.5 mM EDTA) treatment and then lysed in radioimmunoprecipitation assay (RIPA) lysis buffer on ice for 30 min. The samples were electrophoresed on sodium dodecyl sulfate (SDS)-polyacrylamide gels and transferred to polyvinylidene difluoride (PVDF) membrane Immobilon-FL (Millipore, Burlington, MA). The membranes were developed using ECL prime or ECL reagents (Amersham, Pittsburgh, PA), and chemiluminescence signals were visualized using Eastman Kodak X-ray films.

### Total RNA isolation and quantitative real-time PCR.

Total RNA samples from isolated cells were extracted using RNeasy minikit (Qiagen, Germantown, MD) according to the manufacturer’s instructions. The cDNA products were then transcribed using an iScript cDNA synthesis kit (Bio-Rad, Hercules, California). The diluted cDNA products were amplified using a LightCycler 480 real-time PCR instrument with LightCycler 480 SYBR green I master mix (Roche, Indianapolis, IN). The specific primers were designed as follows: for CXCL2, 5′-CGCAGCAGGAGCGCC-3′ (forward) and 5′-TGGATGTTCTTGAGGTGAATTCC-3′ (reverse); for CXCL10, 5′-GAAATTATTCCTGCAAGCCAATTT-3′ (forward) and 5′-TCACCCTTCTTTTTCATGTAGCA-3′ (reverse); for IFN-β, 5′-AGCAGTTCCAGAAGGAGGAC-3′ (forward) and 5′-TGATAGACATTAGCCAGGAGGTT-3′ (reverse); for IL-6, 5′-TGATGATTTTCACCAGGCAAG-3′ (forward) and 5′-AAAGAGGCACTGGCAGAAAAC-3′ (reverse); for IL-18, 5′-GCTCTGTGTGAAGGTGCAGTT-3′ (forward) and 5′-TAATTTCTGTGTTGGCGCAGT-3′ (reverse); for STAT-1, 5′-ATTACTCCAGGCCAAAGGAAGCAC-3′ (forward) and 5′-AGCAAGGCTGGCTTGAGGTTTG-3′ (reverse); for PKR, 5′-TACGTGTGAGTCCCAAAGCA-3′ (forward) and 5′-ATGCCAAACCTCTTGTCCAC-3′ (reverse); for HMGB1, 5′-TACGTGTGAGTCCCAAAGCA-3′ (forward) and 5′-ATGCCAAACCTCTTGTCCAC-3′ (reverse); for IFIT1, 5′-GATCTGGAAAGCTTGAGCCT-3′ (forward) and 5′-GGGTGCCTAAGGACCTTG-3′ (reverse); for GAPDH, 5′-GAGGACAGAGACCCAGCTGCC-3′ (forward) and 5′-TGGAATTTGCCATGGGTG-3′ (reverse). IFN-κ primers were obtained from Qiagen. All quantitated signals were normalized to GAPDH levels in the same cell backgrounds, and the representative data are from three independent experiments. Significance was determined using Student’s *t* test, and a *P* value of <0.05 was considered significant.

### Southern blot analysis.

Keratinocytes were induced to differentiate in high-calcium media for 72 h. Total DNA was extracted using phenol-chloroform according to the standard protocol. The purified DNA samples were then assayed by Southern blot analysis as previously described (18). Total DNA levels were measured by multiple nanodrop measurements using a Nanodrop2000 (Thermo Scientific, Waltham, MA). Equal loading of gels (5 μg DNA) was confirmed following electrophoresis by ethidium bromide staining.

### Lentiviral virion production and transduction.

Mission short hairpin RNA (shRNA) lentiviral vectors expressing five ATR or p62-specific shRNAs (constructs 1 to 5) were purchased from Sigma (Sigma-Aldrich, St. Louis, MO). The vectors were packaged into capsids in 293T cells, and lentiviral particles were prepared as previously described (44). CIN612 HPV-positive cells were then transduced with these lentiviral soups overnight at 37°C. The viral soups consist of concentrated shRNA or scrambled shRNA lentiviruses and 4 μg/ml hexadimethrine bromide (Polybrene; Sigma-Aldrich, St. Louis, MO) in 5 ml E medium. The incubated cells were changed to fresh E medium the next day for an additional 48 h before further processing or analysis. Stable knockdown lines were selected by coexpressed puromycin drug resistance markers.

### RNA-seq analysis.

Total RNA was extracted using RNeasy minikit (Qiagen, Germantown, MD) according to the manufacturer’s instructions. The samples include the cells transduced with the scrambled shRNA (labeled TRC) and the ATR knockdown (shATR) cells with (diff_0) or without differentiation (diff_72). TRC was used in the RNA-seq assay by the investigators performing the assays to refer to the scrambled control. Next-generation sequencing was performed at the Center for Research Informatics, University of Chicago, using Illumina HiSeq 4000 NGS platform. As input to CRI Illumina RNA-seq pipeline, the sequencing data were used for read mapping, postalignment quality control (QC), and expression quantification, as well as identification of differentially expressed genes (DEGs). FastQC v0.11.5 (45) was used to access the quality of raw sequencing reads, and RSeQC (46) and Picard tools v2.9.0 (http://broadinstitute.github.io/picard/) were used for evaluating the postalignment QC. Reads were mapped to GENCODE human genome model (GRCh38) using STAR v2.5.2b (47). Gene transcripts were assembled and quantified using count-based method featureCounts (48). Differentially expressed genes were identified with edgeR (49, 50) by comparing the duplicate samples in the knockdown group to the duplicate scrambled control samples. Finally, functional enrichment analysis was performed with Qiagen’s Ingenuity Pathway Analysis (IPA). The work of TopBP1 knockdown RNA-seq has been published in our previous work (24).

### Data availability.

For the results of the RNA-seq analysis, the GEO accession number is GSE142482. The sequencing data can be found at https://www.ncbi.nlm.nih.gov/geo/query/acc.cgi?acc=GSE142482.
