# Supplementary figures and images for: Pathogenesis of Human Papillomaviruses Requires the ATR/p62 Autophagy-Related Pathway
Source: mBio. 2020 Aug 12;11(4):e01628-20. doi: 10.1128/mBio.01628-20 (PMC7439466; doi:10.1128/mBio.01628-20)

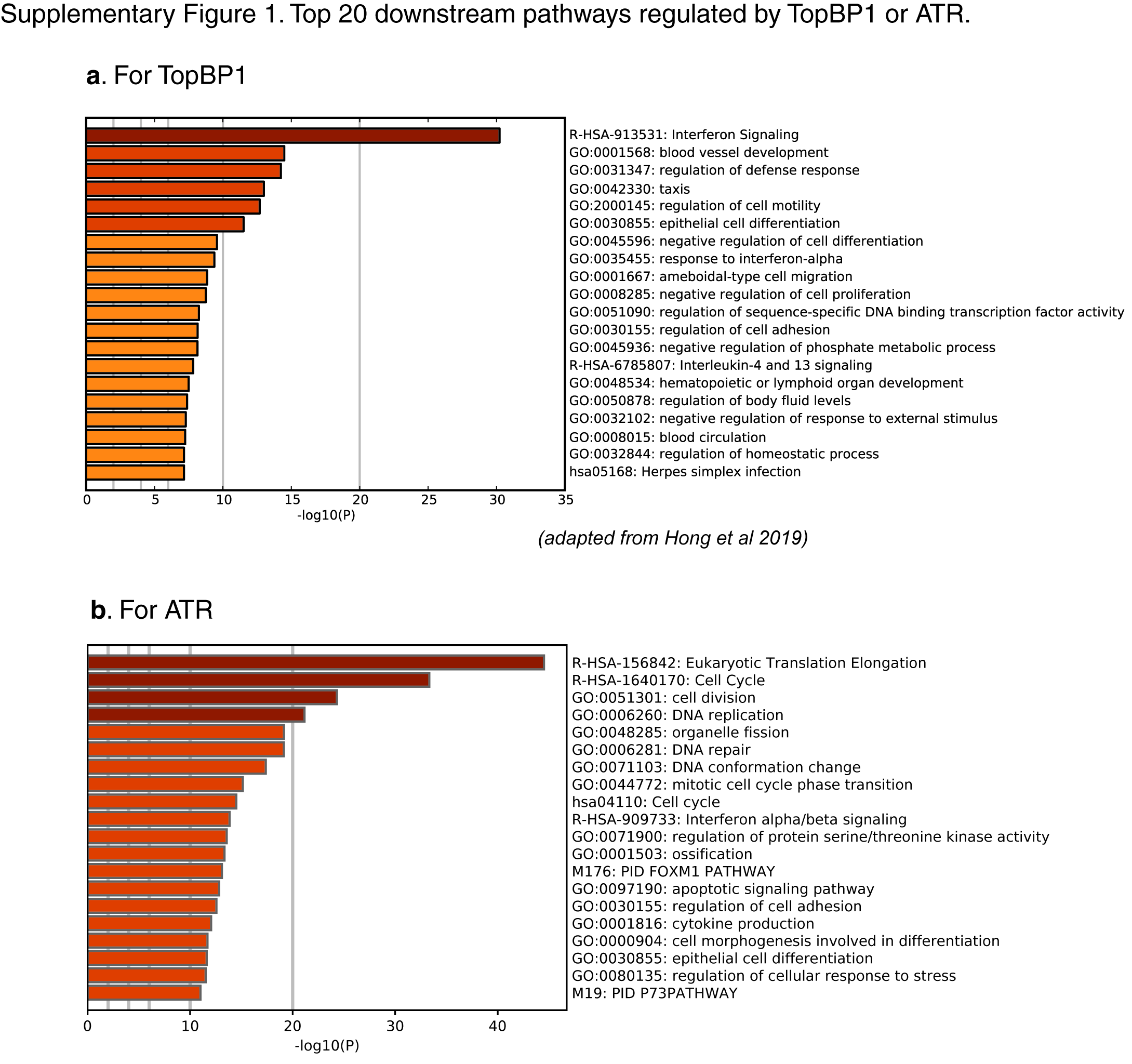

Supplement: FIG S1 [file mBio.01628-20-sf001.tif]

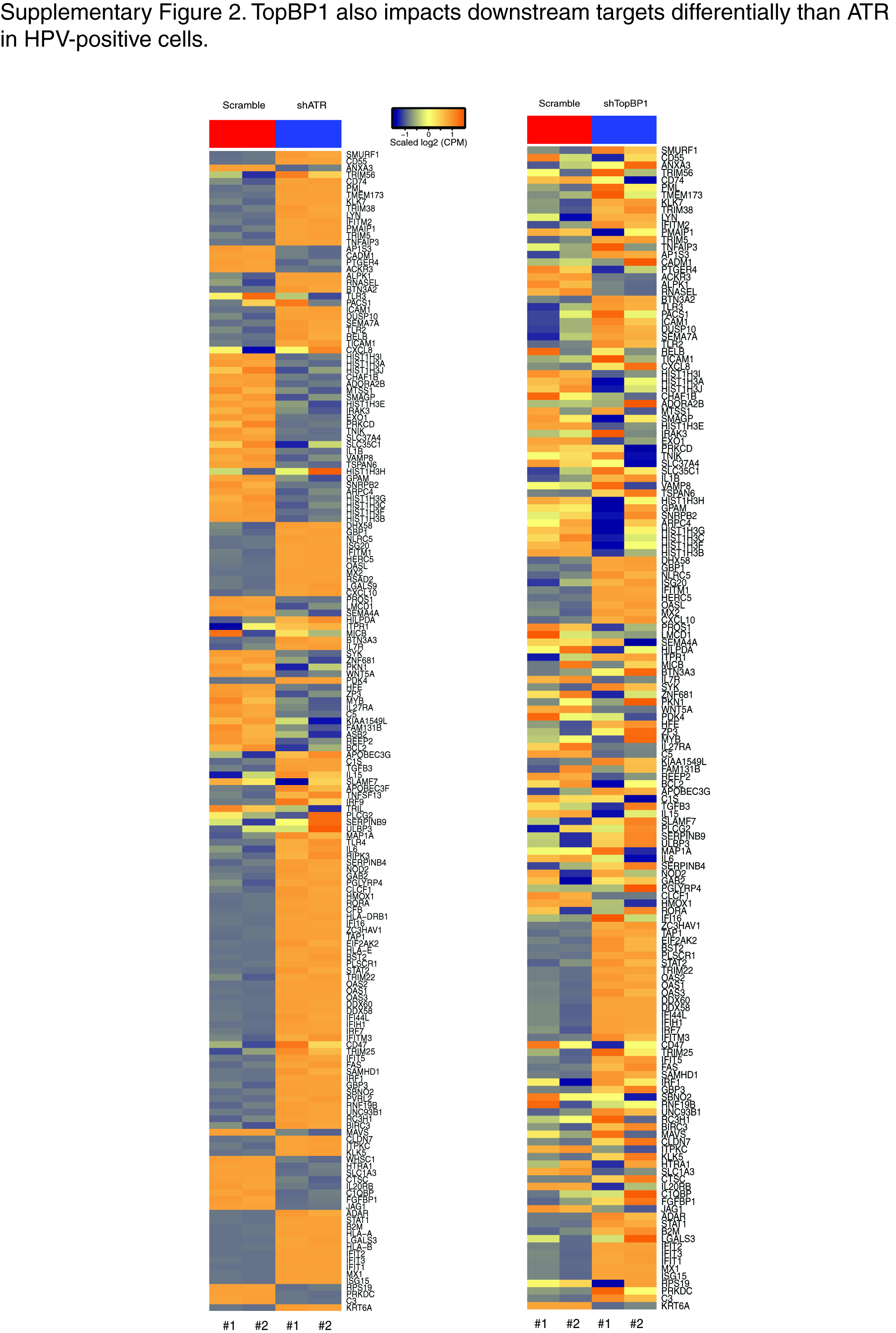

Supplement: FIG S2 [file mBio.01628-20-sf002.tif]
